# Down syndrome: Parental experiences of a postnatal diagnosis

**DOI:** 10.1177/17446295221106151

**Published:** 2022-06-14

**Authors:** Fiona Mc Grane, Fiona Lynn, Joanne Balfe, Eleanor Molloy, Lynne Marsh

**Affiliations:** School of Nursing and Midwifery, 1596 Queens University, Belfast, Ireland; Department of Paediatrics, 8809 Trinity College, Dublin, Ireland; Department of Neurodisability and Developmental Paediatrics, 575353 Children’s Health Ireland at Tallaght, Dublin, Ireland; School of Nursing and Midwifery, 1596 Queens University, Belfast, Ireland; Department of Paediatrics, 8809 Trinity College, Dublin, Ireland; Department of Neurodisability and Developmental Paediatrics, 575353 Children’s Health Ireland at Tallaght, Dublin, Ireland; Neonatology, Children’s Health Ireland at Crumlin, Ireland; 275542 LauraLynn Ireland’s Children’s Hospice, Ireland; Department of Paediatrics, 8809 Trinity College, Dublin, Ireland; Department of Neurodisability and Developmental, Paediatrics, 575353 Children’s Health Ireland at Tallaght, Dublin, Ireland; Neonatology, 8830 Coombe Women’s and Infant University Hospital, Ireland; Neonatology, Children’s Health Ireland at Crumlin, Ireland; School of Nursing and Midwifery, 1596 Queens University, Belfast, Ireland

**Keywords:** Down syndrome, postnatal diagnosis, parents, intellectual disability

## Abstract

Globally it is estimated that Down syndrome occurs in 1 in 800 live births (Bull 2020). It has also been estimated that the incidence of Down syndrome occurs in 1/444 live births in the Republic of Ireland. Given the prevalence of Down syndrome births in Ireland and the fact that care is provided by the majority of parents at home, this qualitative study aimed to explore the experiences of Irish parents receiving a postnatal diagnosis of Down syndrome. A qualitative research approach was used through semi structured interviews. Eight parents of a baby diagnosed postnatally with Down syndrome participated in this study sharing their stories of their postnatal diagnosis experiences. Five overarching themes emerged using a descriptive thematic analysis; 1. prenatal screening, pregnancy and delivery; 2. how the diagnosis was delivered; 3. setting and emotional experiences; 4. moving on with the postnatal diagnosis and 5. Future recommendations from parents' perspectives. This study highlighted the importance of the need for clinicians to ensure that partners are present at the time of the disclosure, that ample time is allocated and that verbal and written communications are provided to parents using less medical jargon when delivering the postnatal diagnosis of Down syndrome. These reasonable adjustments could alleviate parental anxiety at this critical juncture in their lives. Online resources and support forums were also identified as an integral support for families on discharge from the maternity centres and in the early months and years.

## Introduction

Down syndrome (Trisomy 21) is a genetic disorder, discovered by John Langdon Down in 1866 (Diamandopoulos and Green, 2018). It is also the most common chromosomal cause of an intellectual disability worldwide (De Graaf et al, 2017; O’Neill et al, 2018) in which a baby has an additional copy of chromosome 21 in the human cell. Although many children born with Down syndrome have little or no health complications, multiorgan involvement is common (Lagan et al, 2020). Currently in Ireland genetic screening by means of maternal blood sampling known as Harmony testing is an elective investigation which is not offered routinely, nor is it without charge financially. The majority of mothers do not receive a harmony test during their pregnancy. Other European countries are recognised as including maternal blood sampling as part of prenatal care pathways. A review by Asim et al. in 2015, reviewed prenatal screening of Down syndrome describing invasive and non-invasive prenatal diagnostic measures. Invasive prenatal screening measures include amniocentesis and chorionic villus sampling, which both pose an increased risk of miscarriage following the procedures. Non-invasive testing measures include a nuchal translucency scan and foetal anomaly imaging. This diagnostic prenatal testing often identifies cardiac/gastrointestinal defects or other soft markers associated with the condition, including nuchal fold thickness, small nasal bone, and small femur length. Postnatal diagnosis on the other hand, is based primarily on the clinical presentation of the baby immediately or within a few hours after birth and subsequently confirmed by karyotyping or microarray (Crombag et al 2020). While clearly research interest in prenatal screening and a diagnosis of Down syndrome continues to expand (Crombag et al, 2020; Lou et al, 2020), fewer studies have focused specifically on the postnatal diagnosis of Down syndrome (Staats et al, 2015). Irrespective of the timing of the diagnosis, prenatal or postnatal, from the moment of that disclosure, parents transition from becoming a parent to being a parent of a child with Down syndrome (Crombag et al 2020), an often unexpected emotional and life-changing experience (Skotko et al, 2009). For many parents the visible and often recognisable features such as the round flat face and slanted eyes is the first indication at birth that the baby has Down syndrome in the postnatal period (Diamandopoulos and Green, 2018). However, a postnatal diagnosis of Down syndrome is often unexpected by these families and results in a change of life for many (Canbulat et al, 2014; Marsh et al, 2018). To further contextualise Irish parents’ experiences, this study focused on the postnatal diagnosis to gain a better understanding of parents’ experiences to inform future policy and future practice.

## Methods

### Design

This qualitative study was conducted with eight Irish parents to explore their experiences of receiving a prenatal diagnosis of Down syndrome.

### Participants and recruitment

Of the 46 of parents who met the eligibility criteria, a total of eight parents consented to participate in the study, of which seven were mothers and one was a father. The inclusion criteria were that the child was four years of age or younger and parents had received a postnatal diagnosis of Down syndrome. Participants were recruited through a gatekeeper, who was a neonatologist/paediatrician from a medical clinic that their child attended in a large Irish children’s hospital. During the initial recruitment by the gatekeeper, parents provided verbal consent to the Consultant Paediatrician for their contact details to be shared with the researcher. All participants were then provided with the study information including a participant information leaflet and a consent form. Eight parents contacted the researcher directly by phone to arrange an appropriate venue and time for the interview to be conducted.

### Participant’s demographics

Seven mothers and one father, all known to the researcher consented to participate in the study and were unrelated. They were parents to children between the age of 7 months and 3 years and 5 months (5 girls and 3 boys) with a postnatal diagnosis of Down syndrome. Each of the parents accompanied their child to the appointment at which they were recruited with a higher number of mothers present. All mothers were not in paid employment or availing of maternity leave at the time of the interviews. Tom was a full time stay at home parent. Two children were less than one year of age and the oldest child was almost 3 ½. Of the children, one was an only child while the other seven children had between one and 3 siblings (see Table 1).

**Table 1.** Participant and Child profile. (Pseudonyms were utilised)

Parents name/relationship to child	Age of child	Name of Child with Down syndrome
Ann (mother)	7 months	Lucy
June (mother)	2 years	Maeve
Maria (mother)	3 years 5 months	Saoirse
Shona (mother)	3 years	Thomas
Tom (father)	2 years 5 months	Joseph
Lydia (mother)	10 months	Lauren
Barbara (mother)	1 year 8 months	Adam
Gina (mother)	1 year 6 months	Sarah-Louise

### Ethics

Ethical approval was sought and granted by the hospital ethical review committee prior to study commencement (2018-10).

## Procedure and data collection

Participants were given the option to have the interview carried out at a mutually agreed date, time and venue. Four interviews were conducted in a private office in the hospital, three were in participants own home and one took place over the telephone. The single interview lasted between 10 and 45 minutes. Verbal reassurance was given to the parents before the interviews began including their right to confidentiality and their right to refuse to take part or withdraw from the study at any stage of the process. Participants were also once again informed of the aims and objectives of the study. The researcher referred to the interview guide on occasion to provide prompts to participants to ensure that the aims and objectives of the study were met. During the interview, participants were at times asked to clarify meaning to some of their responses through probing questions. These steps were taken to limit potential bias as participants were known to the researcher and establishing a rapport and acknowledging that asking these questions could be emotive. Each interview was recorded and transcribed verbatim with participant’s consent. Field notes were also taken and reviewed and reflected upon. Pseudonyms were assigned to participants and their children to further ensure anonymity.

## Data analysis

In this study, a thematic content analysis (Newell & Burnard, 2011) was used to analyse parents shared stories. Data analysis took place both during and after the research interviews. Interviews with consent, were audio recorded and each taped interview was transcribed directly after the interviews to allow the researcher to be familiar with the information and immerse herself in the data.

## Results

Five overarching themes and a number of subthemes emerged from parental experiences of a postnatal diagnosis of Down syndrome (Table 2).

**Table 2:** Themes and subthemes

Theme 1: Prenatal screening, pregnancy and delivery ○ Prenatal suspicions ○ Referral to geneticist ○ Screening options ○ Visible signs of Down syndrome
Theme 2: How the diagnosis was delivered ○ Positive disclosure ○ Uncertainty ○ Medical terminology
Theme 3: Emotional experiences of the disclosure ○ Setting -Delivery room with partner; Postnatal ward at night and alone ○ Emotional experiences
Theme 4: Moving on with the postnatal diagnosis ○ “He is my son”- coping ○ Questioning and information seeking ○ Online parent forums
Theme 5: Future recommendations from parent’s perspectives ○ Positive and compassionate disclosure ○ Multiple conversations

### Prenatal screening, pregnancy and delivery

At the beginning of the interviews, participants were asked to share their stories of being informed of their child’s diagnosis. Three parents began describing the prenatal suspicions, at the 20 week anomaly scan that could have been ‘soft markers’ of Down syndrome.

*“At the 20-week scan there was there was a thing with the kidneys, and I Googled it and seen it was a soft marker for Down syndrome” (Maria)*.

*“At the twenty week scan they said we notice the limbs are short, they thought that he had a small femur” (Shona)*

Subsequently Maria, Shona and Barbara were reviewed by the consultant obstetrician. One parent, Barbara was also referred onwards to a genetic counsellor for further investigations following the twenty-week scan. While none of these mums were given a formal diagnosis prenatally of Down syndrome, all were offered reassurance that their child being born with Down syndrome was highly unlikely. One parent was offered further prenatal diagnostic testing, with an amniocentesis, but declined the invitation. Another parent was not offered any further screening but had expressed during the consultation that they didn’t want any further testing carried out. Gina recalled that while she was offered reassurance by her GP no further screening was offered as expressed in the following narrative

*“We were delighted to get pregnant on honeymoon because we had previously gone through IVF with a negative outcome. We decided against prenatal tests because we felt we weren’t going to change anything, we didn’t want to spend the pregnancy worrying, so when the scans were good, and her heart was good too we didn’t worry. I just remember looking at her eyes and going gosh they are different to mine and the shape of them”* (Gina).

Many parents started their stories with the delivery of their baby and their instinct that there may be a problem because either the baby had not been handed over to them immediately following the birth, the visible signs of Down syndrome or the unusual responses from the medical team as recounted by Tom, June and Maria.

*“We had a great pregnancy everything went well until the final week”. “At the delivery there was obviously a big crowd there because we were having twins, he came out lovely, I didn’t notice anything, I was thrilled they were born. My wife started to get worried something was wrong as the babies weren’t with us, when they all came in (the medical team) we knew that something was wrong”* (Tom).

*“I think I knew straightaway; her neck was thick, and she was quite floppy”.*

*“She the midwife kept counting her toes multiple times and I was kinda like what’s wrong with her? What’s wrong with my baby?”*

### How the diagnosis was delivered

For many of these parents, and following the birth, their recollection of how they were informed was vivid and who was involved in delivering the news was equally as vivid. Five of the eight parents recalled the information being delivered in a positive manner, which was a welcome finding.

For June it was the paediatrician who delivered the postnatal diagnosis:

“The *paediatrician sat down with us, gave us time and went through things with us she was very, very nice”* (June)

While Lydia didn’t explicitly recall who provided the diagnosis, there was an acknowledgement in her story that this disclosure was not easy for them either.

*“I don’t know what the appropriate way is, but I don’t feel like they did anything wrong. I could see that it wasn’t easy for them either, but we all managed in the situation the best we could”.* (Lydia).

Even though June overall had a positive experience, for her the experience was tinged with some negative experiences. When the official diagnosis was given she was happy in the way it was delivered, yet she felt that until the doctors were available to come to the room to deliver the news that there was a lot of uncertainty as captured in the following narrative.

*“The midwife kept speaking loudly and asked to see photos of my older son. It’s the whispering in the corner stuff I thought she was going to die that’s your ultimate fear”. Nobody would actually tell us anything I just turned around and said will somebody tell us? You are thinking is there something like seriously, seriously wrong with her? Is she going to die? I just lost it then the paediatrician came down and I was like get on with it”. (June)*

Gina and Barbara reported that doctors had used medical terminology at the time of diagnosis which was unfamiliar to them and added to their frustrations at such an emotional juncture in their lives as evident in the words expressed.

*“They took her back from me and started acting weird and said they suspected that she had T21 and that drives me mad using that term, we didn’t know what T21 was” (Gina)*

“The *neonatologist asked did we notice anything about the baby. Then he said he believed that he had T21. I remember my husband saying out loud at least he doesn’t have Down syndrome… I shouted at him and told him he did” (Barbara)*

Some participants recalled that the amount of information received was almost too much and too medically focused.

“*The subsequent level of information is crazy, the doctors speaking in medical terms it made my head spin. Too many people came into us I think just the paediatrician should come and talk to you it’s too intrusive”.* (Gina)

“*I have memory of them giving us a pack on Down syndrome and also a black and white sheet of all the medical problems that she could have. I remember thinking are you for real? This is my baby I just grew her; she is a living person and that was cold and very overwhelming”. (Gina)*

This view was reiterated by June and Lydia

“We received purely negative information that she could have this or that wrong [the baby], you don’t have time to register the fact that you have just had a new baby”. (June)

“… *wanted time to go away and relax and take the news in by myself for a little while, I didn’t want to know about the medical things at this special moment”. (Lydia)*

As can be seen across stories, the majority of the parents felt that the amount of medical information they received after the diagnosis was too much which caused further distress with little time allowed to process their new baby’s diagnosis.

### Emotional experiences of the disclosure

All mothers were present in the maternity ward when they informed of the diagnosis of their baby’s Down syndrome. The majority of parents recounted how they had received the diagnosis in the delivery room from the paediatrician while two parents were told in the postnatal ward during the night whilst they were alone.

“They *apologised for telling me on my own, but it didn’t mean anything because it was quite brutal*” (Ann)

The use of the single word ‘brutal’ was extremely powerful in Ann’s story and escalates the effects of receiving this disclosure when alone and during the night.

The emotions associated with receiving a postnatal diagnosis was a shared negative experience for the majority of parents with their ‘worlds being turned upside down’.

“*When it was verbally confirmed your whole world turns upside down there and then. You nearly grieve the child you thought you were going to have even though you have a child, it’s horrible to say, she is here, she is amazing but the life you have for them I suppose is gone to some extent*”. (Ann)

An emotional rollercoaster was described by some of the parents as Tom recalled “*going from high to low”.*

Gina voiced there was “nothing” they could do:

“*Emotionally you go through the whole rollercoaster, but there was nothing we could do she was our little bundle”. (Gina)*

Similarly, Barbara, remembered denying the diagnosis and being emotionally overwrought:

*“Screaming No, I was devastated, I hated it”*

Experiences of fear, shock and blame also resonated across some parent’s stories. For example, Tom, June, and Maria vividly recalled fear of the unknown being a prominent emotion when the diagnosis was given to them.

*“The fear too, I was thinking that he is not going to play football not going to do this not going to do that. He was always going to do what he was going to do”. (Tom)*

*“We were pretty terrified; we were crying” (June)*

The shock of receiving a postnatal diagnosis was also evident in Maria’s narrative.

*“Shocked really shocked and I cried a lot, I just cuddled her and cried” (Maria)*

Ann and Gina initially blamed themselves for their child being born with Down syndrome and questioned their role in their child’s diagnosis

*“Was it something that I did during the pregnancy?” (Ann)*

Gina echoed these sentiments:

*“I was like god… is this all my fault? Am I not going to connect with her? Did I give her Down syndrome?” (Gina)*

Time to question, understand and reflect on the postnatal diagnosis for many parents would have been beneficial as there was little time to transition from being a parent to finding found out they were now parents of a baby with Down syndrome

### Moving on with the postnatal diagnosis

Parents reported that after the initial negative emotions experienced that they began to cope with the diagnosis and their focus changed to the love that they felt for their child, seeking information from a range of healthcare professionals as well as a focus on the future.

“*I think I cried for the initial 5 minutes and then I was like ok he is my son; we didn’t have time to sit around and feel sorry for ourselves we have to step up and look after him. I was like this is my boy we are going to do whatever we have to do to get him through this*”. (Tom)

“*Different Paediatricians were coming and explaining things and I was asking questions… this was amazing” (Lydia)*

*“The social worker told us about early intervention services and the other appointments, and she went through all the forms. She did a lot of the follow up and explained things to us” (Ann)*

Parents also highlighted the support they received from online parent forums and expressed that maternity centres should provide information to parents about such online parent forums or even Facebook.

*“Another thing that would have been helpful was something that the medical staff probably didn’t know was about the Facebook groups they have been super helpful”. (Gina)*

### Future Recommendations from parents’ perspectives

When parents were asked specifically to share advice to healthcare professionals in order to improve the disclosure process, and drawing from their personal experiences, they initially reiterated that they would like the diagnosis to be delivered in a positive manner and with compassion.

“*Don’t come in with a list of negatives, all the negatives that were put in your head initially we never expected her to be this good now. Keep things positive and give the opportunity to ask questions. Be straight, open and honest with parents and give an opportunity to ask questions”*. (June)

*“People should never say a baby cannot do something, because they don’t know”.* (Gina)

“*Doctors should show compassion when they are delivering the news, have patience and don’t be rushing in and out” (Tom)*

For others, advice in relation to timing at which the diagnosis should be given to parents varied.

“*Parents shouldn’t be left in limbo but it’s nice to give a new mum time afterwards to get to know your baby before saying anything” (Maria).*

“*Maybe leave it a few minutes to give the diagnosis let them enjoy their baby” (June)*

Leaving time for parents to process this type of disclosure was also supported by Tom:

“*I wouldn’t recommend that you give the news five minutes after you hold your child for the first time, them moments are magical and precious*” (Tom)

Additionally, many participants felt that it was vital that, at the time of diagnosis, the mother needed to have their partner or a family member present to support them.

“*Don’t give the diagnosis when she is on her own. Like even if she doesn’t have a partner or husband, maybe a sister, her mum or dad make sure there is someone in the room”. (Ann)*

“*Be told with their partner. If the partner can’t be there have someone in the room with them their mother, sister or a friend “. (Shona)*

All parents felt that it was important that maternity centres provided families with information in relation to support services that are available in the community including other parents with children with Down syndrome.

*“It is very important that maternity hospitals are aware of the supports available in different areas*”. (Maria)

*“I didn’t know anyone with a child who had Down syndrome so I would have liked to talk to someone you know” (Barbara)*

## Discussion

The significant emotions experienced with a postnatal diagnosis resonated across these parents’ stories and resonates across many other international studies (Schimmel, et al 2020; Staats et al, 2015), suggesting that this is not just a unique experience to the Irish context. Rather, it reinforces the evidence that parents experience a wide range of emotions irrespective of receiving a prenatal or post-natal diagnosis of their child’s Down syndrome (Farkas et al, 2019; Pillay et al, 2012). Many parent’s preconceived aspirations for their new born were initially shattered at the time of the diagnosis and adequate emotional and psychosocial support was not routinely provided to parents to deal with their own emotions (Griffin et al. 2019). Therefore, the way in which healthcare professionals’ disclose this diagnosis needs to be compassionate, tailored, kind and balanced (Sheets et al, 2011b; Marshall et al. 2019). A welcoming finding in this study was that the majority of parents were satisfied with the way the diagnosis was delivered, a finding echoed by Israeli mothers in a quantitative study with 45 mothers who received a postnatal diagnosis of Down syndrome (Schimmel, et al 2020).

All parents interviewed in this study recommended that the diagnosis is ideally given with a partner or family member present, similar to Israeli mothers (Schimmel et al. (2020), Indian parents (Rasendrakumar et al. 2021) and Irish parents (Smith et al. 2018) of children with Down syndrome. Being informed of the diagnosis alone or during the early hours of the morning, left parents feeling distressed, a finding highlighted across studies. Contrary to the Irish National Federation of Voluntary Bodies Guidelines (2007) a disclosure of a child’s disability should be family centred, with both parents present to promote emotional wellbeing and hope, a further recommendation across other best practice statements (Shazali et al. 2018, Sheets et al, 2011a). Ideally, healthcare professionals are required to be knowledgeable, empathetic, caring practitioners and support parent’s ample time to ask their questions over time. A disclosure should never be considered a one off event, rather it is a process parents are required to navigate (Marshall et al. 2019). To improve the parental experience at the time of disclosure, information should be of consistent quality, timely and accurate (Marshall et al. 2019; Sheets et al, 2011a)

Parents in this study also felt that it was vital that once the disclosure was shared, they should have been provided enough time to ask healthcare professionals’ the questions that mattered to them, a similar finding across international studies (Ahmed et al., 2015; Mc Anuff et al., 2014). Ranjan et al. (2015) suggested that time pressures led to clinicians prioritising their attention to delivering the diagnosis rather than given due consideration to the type of communication and language used. For example, three parents in this study did not recall receiving any written information following the disclosure and were dissatisfied overall with their experience. These findings resonate with parents in Ahmed et al’s (2015) study in which few of the 30 Pakistani parents recalled receiving any written information about Down syndrome. Rather verbal information only was provided at the time of diagnosis which arguably impeded their understanding of their child’s Down syndrome. Providing and receiving printed information was equally recommended by parents and genetic counsellors in an online survey seeking to explore what information was essential at the time of a disclosure of Down syndrome (Sheets et al, 2011a). Sheets et al (2011a) also highlighted the importance of information that was balanced and tailored to the information needs of the parents and devoid of medical jargon would be more satisfactory for parents. While Ranjan et al’s (2015) study was not specific to a disclosure of Down syndrome, the basic principles of practicing good communication such as using effective skills, being attentive and present, being empathetic and reading the non-verbal signs accurately were provided. More importantly, providing balanced and hopeful information about the nature, course and prognosis is important and consistently identified across studies in relation to the disclosure of Down syndrome (Sheets et al, 2011a, 2011b). Therefore, allocation of sufficient time and the provision of written information may play a key role in delivering a diagnosis as well as relieving parental anxiety (Ahmed et al 2015).

Yet, many parents continue to report dissatisfaction with some healthcare professionals’ approach with the disclosure of Down syndrome, a finding also borne out in this current study. An evaluation of training for healthcare professionals such as midwives, paediatricians and health visitors about Down syndrome by Bryant et al (2016) in the UK, reported staffs’ knowledge of Down syndrome and confidence in communicating about DS, including at the time of diagnosis has increased following training.

Even though best practice guidelines to support a disclosure of Down syndrome exists in Ireland (National Federation of Voluntary Bodies (FedVol) (2007) and the United States of America (USA) (Sheets et al, 2011a), parents dissatisfaction experiences with this disclosure continues even though training for healthcare professionals has been consistently recommended in how best to deliver the news (Bryant et al. 2016, Marshall et al. 2019). As part of this training, healthcare professionals should reiterate this information over time and at multiple consultation visits to ensure that parents understand the information required to support their child across the trajectory of caring.

Additionally, the importance of online information sources and parent support forums was also described across studies (Jacobs et al. 2016) with parents in this current agreeing that online support was of great benefit and a key source of information.

This study generated rich narratives adding to the wider body of disability and family research. Some limitations to this study were evident in that the sample size was small and only one of the 8 participants was a father and mothers voices continue to be more prominent. Even though there is an increasing interest in fathers’ experiences, fathers still continue to be less featured in the research of parents’ experiences (Marsh et al, 2018; Ridding and Williams 2019). All of the children with Down syndrome were generally in good health and were accessing a specialist medical clinic which needs to be considered in future research as the experiences with a medically or behaviourally compromised child may generate other insights of being a parent of a child with Down syndrome. This study findings are from the experiences of eight parents and do not claim to be representative of all parents receiving a post-natal diagnosis of Down syndrome, as every experience is so unique.

## Conclusions and Recommendations

The postnatal diagnosis of Down syndrome is an unexpected occurrence for parents and is associated with initial negative experiences of shock and guilt, a similar finding across studies. The importance for healthcare professionals to ensure that partners are present at the time of the disclosure needs to be embedded in policy and practice. A further practice recommendation must highlight how important it is for ample time to be allocated to parents at the postnatal diagnosis of Down syndrome disclosure alongside less medically focused verbal and written communications. Additionally, in this digital age, online resources and support forums are integral in supporting parents from the diagnosis journey and across the lifespan and is an easily accessible platform for parents to receive emotional support and advice from other parents in similar situations. Therefore, healthcare professionals have additional roles and responsibilities to signpost parents to reputable and safe online platforms.

From an education perspective, it is equally evident that a disclosure of Down syndrome is not a one-off event and healthcare professionals require further education about disclosing a diagnosis of a child’s disability. Such a diagnosis is just the beginning of these parent’s journey in which healthcare professionals are well placed to support parents and the wider family.
